# Disease X and COVID-19: turning lessons from India and the world into policy recommendations

**DOI:** 10.1097/MS9.0000000000002391

**Published:** 2024-07-19

**Authors:** Mainak Bardhan, Ishita Ray, Shubhajeet Roy, Priyanka Roy, Priya Thanneeru, Anu Radha Twayana, Sakshi Prasad, Mayukh Bardhan, Ayush Anand

**Affiliations:** a Division of Bacteriology, ICMR-National Institute of Cholera and Enteric Diseases, Kolkata; b Regional Institute of Medical Sciences, Imphal; c Mahatma Gandhi Memorial Medical College, Indore; d Faculty of Medical Sciences, King George’s Medical University, Lucknow; e Chief Inspector of Factories/ Deputy Director (Medical) and Certifying Surgeon, Directorate of Factories, Department of Labor, Government of West Bengal; f Gandhi Medical College, Secunderabad, India; g Kathmandu University School of Medical Sciences, Dhulikhel; h B. P. Koirala Institute of Health Sciences, Dharan, Nepal; i Miami Cancer Institute, Baptist Health South Florida, USA; j National Pirogov Memorial Medical University, Vinnytsya, Ukraine

**Keywords:** Disease X, COVID-19, SARS-CoV-2, zoonotic diseases

## Abstract

Disease X is caused by pathogen X, an unknown infectious agent that can potentially trigger an epidemic or pandemic. Pathogen X might be any pathogen, including bacteria, viruses, parasites, fungi, and prions. WHO uses the term ‘Disease X’ for any new emerging disease caused by an unknown pathogen X. Disease X stands for any possible future pandemic in WHO’s shortlist of high-priority diseases. This review looks at the manifestations of the recent COVID-19 epidemic as the first Disease X to evaluate what has happened and to learn from what went wrong in India and worldwide. To this end, a summary is presented of response measures by governments, often lacking flows of information, discrepancies in the views of experts and decisions of policymakers, and undesirable variations in individual and collective behavior and their consequences. The elements of combating Disease X in a world with considerable inequalities in relevant knowledge, expertise, information, quality of governance, and financial possibilities are discussed. Based on this, recommendations are given for an innovative global pandemic preparedness system.

## Introduction

Highlights

- WHO uses the term ‘Disease X’ for any new emerging disease caused by an unknown pathogen X.
- The lack of international cooperation, unequal distribution of resources, censorship of exact data regarding case numbers and deaths, leniency in social distancing measures, and misinformation can lead to the uncontrolled spread of disease X globally.
- Further studies are warranted to study the spillover of viruses and other microbes from animals to humans and the development of mRNA vaccines.

According to the WHO, ‘Disease X’ represents the knowledge that a serious international epidemic could be caused by a pathogen currently unknown to cause human disease^1^. ‘Pathogen X’, an infectious agent that is presently unknown but has the potential to produce an epidemic or pandemic, causes Disease X. Any pathogen, including viruses, bacteria, fungi, parasites, and prions, could be pathogen X. The WHO’s research and development blueprint was launched in 2016 to shorten the time it takes to discover, evaluate, and approve medicinal countermeasures for the world’s most lethal pathogens^2^. Disease X was a term the WHO chose in February 2019 to signify a future pandemic on their shortlist of high-priority diseases.

The knowledge of Disease X promotes the focus of research efforts on entire classes of viruses (e.g. Flaviviruses) rather than single strains (e.g. Zika virus), improving their capacity to act to unforeseen strains^3^. It is recommended that the WHO and competent health authorities keep a watch on lately increased incidences of infectious illnesses such as Ebola, Zika, and Dengue, among others, since they have the potential to create devastation comparable to the present COVID-19 epidemic^4–8^. COVID-19 is caused by the novel SARS-CoV-2 virus and transmitted via three primary modes known as ‘contact’, ‘droplet’, and ‘aerosol’ transmission^9^. Thus, with each epidemic or pandemic, global health services have improved preparation, management, and risk communication strategies, emphasizing the importance of taking appropriate action before the complete clinical profile and etiological agent behind the new disease are fully understood^10^.

## Candidates of disease X and its clinical forms

Disease X holds the potential to outbreak as an epidemic or a pandemic. It is imperative for the public health department to list emerging infectious diseases to promote accelerated vaccine development^2^. To raise awareness and stimulate research, the WHO convenes an expert committee yearly to update its list of extremely serious infectious illnesses with no viable treatments or vaccines. The current list contains Rift Valley fever virus, Zika virus, MERS-CoV (Middle East Respiratory syndrome coronavirus), Marburg virus disease, Ebola virus disease, Crimean-Congo hemorrhagic fever virus, Lassa fever, severe acute respiratory syndrome (SARS) virus, Nipah virus, Henipaviral disease and Disease X^11^.

During an annual review in 2017, WHO added the Zika virus to the list after causing an unexpected rise in cases of microcephaly^12^. The WHO announced in 2019 that the Wuhan pneumonia of unknown etiology should be recognized as the first Disease X^13^. The first Disease X now has a name, COVID-19, which was added to the list of conditions for research prioritization^13,14^.

RNA viruses are more likely to be the infective viruses for newly emerging disease X due to their high potential to mutate into variant forms. The causative pathogen is not limited to viruses but includes bacteria, viruses, fungi, parasites, and prions. The zoonotic reservoirs of pathogens use insect vectors to infect the human population^15–23^, as cited by Simpson *et al*.^2^. For instance, the outbreak of the H1N1 virus consisted of the genetic material of avian, human, and swine origin involving wildlife and migration between animals and farm workers. The spillover of animals to humans in SARS-CoV-2 was not apparent, but the mutations in animals before transmission to humans have been supported for the emerging threat^24^. A possible bat origin was suggested by Zhou *et al*.^25^ and cited by Jiang *et al*.^13^, after the whole genome sequence of the novel coronavirus SARS-CoV-2 matched with bat SARS-related coronavirus (SARSr-CoV-RaTG13) by 96%.

**Figure 1:**
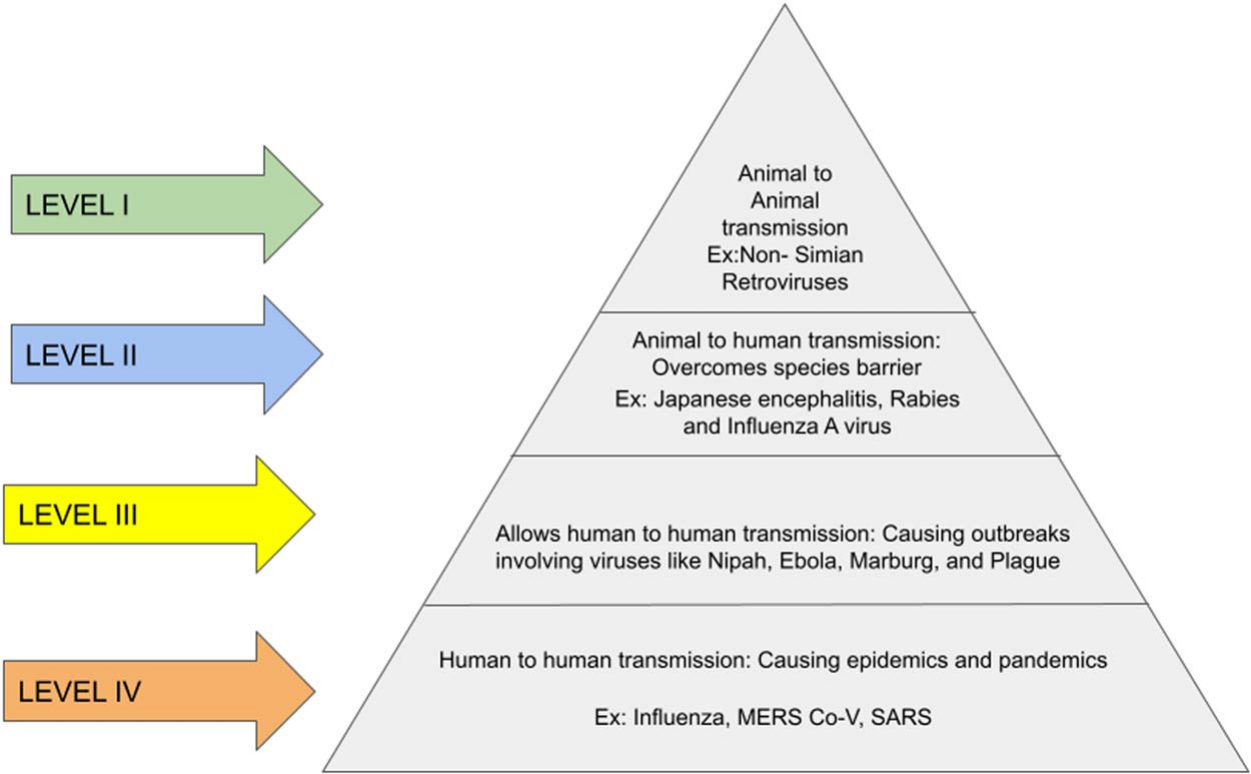
Pathogen Pyramid Model to understand the origin of zoonotic diseases in humans. MERS Co-V, middle eastern respiratory syndrome coronavirus; SARS, severe acute respiratory syndrome.

As cited in Chatterjee *et al*.^26^, the ‘Pathogen pyramid’ model has been used to understand the origin of zoonotic disease in the human population (Fig. 1). Four levels have been categorized to understand the origin and to group the severity of different origins. Level 1 includes direct animal-to-human transmission, mostly involving non-simian retroviruses. Level 2 represents zoonotic diseases overcoming species barriers to infect human cells but holds the slightest chance of transmission from human to human. Examples are the Japanese encephalitis virus, rabies, and influenza A virus. At level 3, the zoonotic pathogens promote transmission from human to human, resulting in outbreaks that blow up and eventually settle down, likely involving viruses like the plague, Nipah, Ebola, and Marburg disease virus. Ultimately, at level 4, human-to-human transmission occurs, leading to epidemics and pandemics such as ongoing COVID-19 and its preceding forms of influenza, MERS-CoV, and SARS. HIV can also be considered at this level, given its epidemiology^18,27–42^.

## COVID-19 as the first Disease X and lesson learned from the pandemic

### *Global impact and what went wrong*:

Diverse strategies were planned and implemented across the globe for the containment of COVID-19. It is almost four and a half years since the virus was first reported from China, and although the pandemic seems close to ending, it is still far from reality. However, it is still possible to map out certain vital lessons we can learn from COVID-19.

On 31 December 2019, the WHO head office was officially contacted for cases of ‘pneumonia of unknown etiology (unknown cause)’ detected in the City of Wuhan, Hubei Province in China, yet according to reports, healthcare officials and scientists had informed the Chinese government bodies weeks earlier of a SARS virus-like illness prevalent among their patients^43^. Rather than creating an alert system to spread awareness to the rest of the province and the higher authorities, they choose to conceal case numbers and pressed charges of spreading false rumors against the physicians who spoke up, most notable being the now late Dr Li Wenliang. Experts have suggested that if appropriate measures were implemented a mere few days in advance, the eventual spread of the SARS-CoV-2 virus could have been lowered considerably^43^.

‘Global solidarity and unity is the sole solution to this war against COVID-19’. This pandemic saw poor international coordination and many powerful countries trying to obtain sole rights to the COVID-19 vaccine without considering middle-income countries and low-income countries.

Attempts at communication and collaboration at the highest inter and intragovernment levels have been significantly disappointing. The first virtual G7 and G20 summits occurred months after the pandemic began. Even when the leaders came together and discussions did take place, there was a lack of consensus among world leaders. When the G7 summit happened in March, instead of discussing coordination and solidarity to fight the pandemic, disputes on which country was to be blamed took place, and a postmeeting joint statement failed to be drafted^44,45^.

### EcoHealth Alliance

*Members of the EcoHealth Alliance have repeatedly raised alarms about the potential risks posed by Disease X, stressing the importance of prevention over the costly aftermath of allowing such diseases to emerge unchecked. As early as 2017, they sounded the alarm about the pandemic potential of SARS-like viruses. The EcoHealth Alliance is affiliated with the Global Virome Project (GVP), an international collaboration aimed at enhancing our ability to prepare for and respond to outbreaks. Their mission is to establish a network of public, private, philanthropic, and civil organizations to identify unknown viral threats and prevent future epidemics. According to their estimates, 71% of Disease X threats could be discovered with a budget of just $1.2 billion, a fraction of the cost incurred in responding to the 2002-‘03 SARS CoV outbreak, which amounted to $30-50 billion. However, as evidenced by the global COVID-19 pandemic, these warnings were largely ignored. Leveraging resources such as the GVP and global databases could facilitate the development of better diagnostic tools and more accessible pathogen detection and sequencing technologies, ultimately aiding in pinpointing and addressing current hotspots. These efforts, combined with advances in bioinformatics and artificial intelligence, hold promise in proactively preventing pandemics through continuous capacity building* ^46,47^.

### International Health Regulations 2005

International Health Regulations (2005) (IHR) provide an exhaustive legal framework defining countries’ rights and obligations in handling major public health events and emergencies that can cross borders. IHR legally binds 196 countries, including the 194 WHO Member States. The IHR was formed to create rights and obligations for countries, like the requirement to report any public health events. The IHR requires that all countries be able to do the following: Detect: Make sure surveillance systems can detect acute public health events in a timely manner. Assess and report: Use the decision instrument of the IHR to assess public health threats and report to WHO. Respond: Respond to public health threats and such emergencies. The goal of country implementation is to limit the spread of health risks beyond borders to neighboring countries and prevent unnecessary travel and trade restrictions. The IHR 2005 could have been crucial in preventing the current COVID-19 pandemic and future disease X pandemics. However, timely reporting and responses to control spread were not made by several member countries. Despite global agreement on the importance of IHR (2005), only a third of the member countries had the logistics to implement the IHR recommendations to assess, detect, and respond to public health emergencies. Until we bridge the gap of technology and resources between nations, the global population will remain vulnerable^48,49^.

### Organization for economic cooperation and development (OECD) policy responses to coronavirus

The OECD synthesized the evidence gathered from 67 member countries that analyzed government responses during the first 15 months of the COVID-19 pandemic. This was done to identify ‘what is working, what is not, what *could* work, and for whom’. Pandemic preparedness was found to be mostly insufficient in most member countries. Crisis communication and overall trust between nations seemed to be lacking. They urged to explore the effectiveness of lockdowns and restrictive measures considering the huge toll they had at an individual level with a negative impact on domestic life, mental health, and increased alcohol and drug consumption^20^.

We need an improved understanding of hazards and public health risks, allowing countries to devise better prevention and mitigation strategies to reduce their vulnerability to a future disease X outbreak. Effective crisis management determines a country’s resilience to pandemics. Evidence from the OECD shows that managing a future disease X emergency involves many actors that go way beyond just emergency services. This requires coordination and creates challenges like governing the different sectors involved. They also study the economic impact of a future pandemic on small businesses, self-employed workers, and vulnerable sections of society, to name a few^50^.

### Call for reforms in the current global pandemic warning systems

Carroll *et al*. implied that current identification means are weak and often set to be missed by classic surveillance systems. Existing capacities, processes, and institutional arrangements, like the IHR and the Global Health Security Agenda, were not sufficient to prevent this, and testimony to this fact is the COVID-19 pandemic. Lessons learned from COVID-19 emphasize the need to create global strategies, policies, and regulatory frameworks that deal directly with the multifactorial aspects of disease emergence and improve the collective ability to prevent, rapidly detect, and respond to threats. Supplementary to all these is the need to create a surveillance system covering humans and animals, whether livestock or the wilderness. There is a need to construct the existing surveillance systems of various designs and unify them all to go towards a multisectorial approach. This will only be possible by adopting standardized protocols and data-sharing commitments^51^.

Tahir *et al*. also expressed the extreme need to develop international protocols to counteract bioterrorism, which might be a possible nidus for a potential epidemic. Next, academic advice must be taken well in advance and in regular interim, without any political influence. A comprehensive method to look into the underlying issues for the spread of Disease X is by bridging institutional gaps, defining priority risk zones and disease-causing species, and highlighting probable risk factors for upcoming suspected events involving emerging and re-emerging diseases of infective pathology^52^.

An aspect that cannot be denied is that during the COVID-19 pandemic, the world has come a long way forward in terms of preparedness for future pandemics. Warning systems can be improvised further based on learnings and feedback from previous pandemics to prepare better versions of the current ones rather than beginning everything from scratch.

### Global inequality in vaccine doses and access to PPEs and vaccine hoarding

The existence of inequality in the current international scenario has led to high-income countries ordering surplus vaccine doses enough to vaccinate their population several times over, whereas low to middle-income countries have not even been able to cover their entire population once due to the shortage of vaccine doses. There also have been reports of colossal vaccine wastage in countries due to excess amounts, opened vials, expiration, vaccination-related hesitancy, and logistic issues. Canada has ordered enough vaccines to vaccinate its population five times over, followed by the UK, The European Union, Australia, and the USA. The Serum Institute of India was a significant player in ensuring equitable access to vaccines by being the largest exporter of COVID-19 doses of its home-manufactured vaccines despite catering to one of the largest populations on the home front. Policies need to be made for equitable vaccine distribution in coming disease X pandemics to achieve global herd immunity faster^53^. The COVID-19 Pandemic has also seen large-scale scarcity and inequitable access to personal protective equipment (PPEs), which formed the basic need for at least those looking after and treating the patients. This in turn, led to an increased proportion of healthcare personnel being hunted for the deadly virus^54^. Hence, policies for equitable distribution of PPEs are also of utmost necessity. Particularly important are the mrNA vaccines representing an evolving modality in COVID-19 mitigation across^55^.

### The situation of COVID-19 in India and the factors contributing to the challenges faced during the second wave of the pandemic

Many reasons contributed to the disastrous second wave of Indian COVID-19, including the interaction of novel mutant strains, failure to follow COVID-appropriate procedures such as mask-wearing, social separation, and other COVID-appropriate behavior, and a delay in completing the vaccination push^56,57^. A new SARS-CoV-2 lineage B.1.617 emerged in India and has been held responsible for the surge in cases due to higher transmissibility of this strain having quickly spread to multiple countries^58^.

Series of political campaigning for state elections in some of the most densely populated states like West Bengal and Tamil Nadu, and religious mass gathering events, including the gathering of ~9 million pilgrims at the Kumbh Mela festival, ended up acting as a catalyst to begin the second wave^58,59^.

Foregoing all the social distancing and lockdown measures for the propagation of political and religious agendas can be held accountable. Hesitancy to take vaccines, concealment of the actual data of the number of cases and deaths, and delays in the vaccination drive added to the case surge^55^.

Public health policymakers were accustomed to less aggressive variants. There was a negligent attempt made after the first wave to study the wave, learn lessons from it, bridge the gaps in the Indian healthcare system, educate the masses to follow the rules still, and most critically prepare for the second wave in a better fashion^58^.

## Recommendations for disease X

There is an imperative need for an innovative global pandemic preparedness system owing to the utter failure of the public health system worldwide during the current COVID-19 pandemic^3^.

- Predicting the pandemic potential of novel microbes: new metagenomic technique can identify potential human infections in other organisms. This contains studies on the significance of host-relatedness.
- Host relatedness: evidence suggests that viruses from more closely related hosts (e.g. humans and chimpanzees) are more likely to spill over from one species to another^60,61^.
- Virus relatedness is another predictor of pandemic potential as viruses in wildlife that are more similar to existing human pathogens are more likely to cause new diseases in people and hence need to be surveilled. The viruses with high mutation rates with no proofreading mechanisms pose a greater risk of infecting people^62^.
- Preventing zoonotic spillover and environmental protection: scientists created a risk rating methodology and an interactive online application called ‘Spill Over’ to predict a vulnerability index for wildlife viruses by comparing the danger of viruses with undetermined zoonotic spillover prospects to viruses that are documented to be zoonotic^62^. In environmentally chaotic places, we need to determine where spillover is most likely. The risk for spillover increases in areas where human habitat comes close to wildlife reserves, as with excess deforestation and habitat loss. The consequent rise in the populations of birds, rodents, and bats in human habitats increases people’s exposure to zoonotic virus reservoirs. Hence, conserving rainforests and other wildlife resources is vital to reducing future pandemics. The spillover of pathogenic agents from other species to humans is extremely unpredictable, forseeing pandemics caused by such zoonosis is consequently an arduous task^20^.
- Prepandemic preparation of medical countermeasures can be crucial: the COVID-19 pandemic showed us how concealing cases or delaying adequate personal protective measures can culminate in tremendous human suffering and death. International Bodies like the WHO need to be immediately alerted to avert global catastrophes. An expanded database of pathogen sequences will help to swiftly triage and identify homologous pathogens to the future pathogen X. Hence, prepandemic medical countermeasures can be prepared regardless of the identity of pathogen X. Developed nations can help expand their diagnostic companies to low-and-middle-income countries to help them establish infrastructure for managing the burden of a pandemic in future. Regulatory measures and paperwork for starting the research on a new pathogen vaccine or small molecule for therapy against future pathogen X need to be fast-tracked. This can be made possible by designating bodies in the government for prepandemic management by allocating funds to research on the pandemic potential of pathogens and preventive measures.
- Integrating Services: following the saying ‘prevention is better than cure’ will never become more relevant than the current times. There have been pandemics in the past, and there certainly will be more pandemics in the future. The only solution is to learn from the mistakes, plan, and attempt to minimize the damage and loss by acting early. We must be prepared for impending pandemics by combining public health, healthcare, and emergency management services. Government and nongovernment entities must work hand in hand to make this possible.
- Sample transportation and diagnosis: logistical difficulties in transporting blood and other serum samples are a huge barrier in expediting diagnoses of future disease X. Storing and transporting such hazardous samples from remote areas is inefficient and can be solved by setting up regional locations’ biorepositories. A biorepository or biological materials repository collects, processes, stores, and distributes biospecimens to support future scientific diagnoses^63^.
- Data-sharing: sharing data on emerging disease X pathogen sequences and lowering the time it takes to form contracts for intellectual property sharing between states or nations might accelerate research on potential treatments or vaccinations for a future pathogen X and save millions of lives. The pandemic Influenza Preparedness Framework is a ground-breaking project for increasing access to vaccinations, diagnostic kits, and antiviral medications, particularly in low-income countries^2^.
- Vaccine development for emerging diseases: vaccination is the best modality to prevent and control infectious diseases^64^. The wonder called ‘mRNA’ vaccines against infectious microbes can be manufactured as therapeutic or prophylactic agents. mRNA vaccines carrying antigens of virulent pathogens induce potent and robust immune responses. The manufacture of mRNA vaccines is a cell-free process, rapid, and straightforward vs other types of vaccines. This makes mRNA vaccines a promising biological tool to cover the gap between emerging pathogens and the dire need for potent vaccines against them. The use of an intramuscular dose of an mRNA-RBD vaccine led to broad-spectrum neutralizing antibodies and cellular responses not limited to the wild-type SARS-CoV-2 virus but also the Delta and Omicron variants^64^. Large-scale RNA production to accomplish commercialization is the pioneering step toward making mRNA vaccines. In 2017, several countries and international organizations started a vaccine preparedness initiative. Germany, Japan, India, Norway, the Welcome Trust, the World Economic Forum, and the Bill & Melinda Gates Foundation established the strategic Coalition for Epidemic Preparedness Innovations (CEPI) to grow support to fight significant health epidemics/pandemic threats through vaccine development^3^. Nanovaccines are another potential resource that can be explored to tackle a future DiseaseX outbreak. Nanovaccines are made of nanoparticles associated with or prepared with components that can stimulate the host’s immune system. They also demonstrate possession of intrinsic adjuvant properties and work as immune cell stimulators. Thus, nanovaccines can promote rapid and prolonged humoral and cellular immunity^65,66^.
- Creating Safety Guidelines and prepreparedness for outbreak management: during a pandemic, people require appropriate leadership and consistency to maintain public morale. As a result, leaders must be prepared to manage healthcare emergencies such as the COVID-19 pandemic. Provide safety precautions. Educating people on how to safeguard themselves and their families is critical. Some examples are social distancing legislation and proper mask etiquette. Travel restrictions and airport screening should be implemented. Support viral containment, restricting virus dissemination to new areas, testing, and active contact tracing. Make a stockpile of all required drugs and equipment. Protect healthcare workers at significant risk for contracting the disease. An adequate supply of personal protective equipment and keeping stockpiles of all necessary medications in advance may become indispensable during a pandemic.
- Development of a global intervention target product profile (iTPP): the required qualities of a new product to meet a high unmet clinical demand are defined by a target product profile (TPP). They guide drug or vaccine manufacturers to develop ‘fit for purpose’ products, thus allowing novel therapeutic or preventive agents to go faster from labs to patients^67–70^.
- Clinical leadership and consistency: they are observable and learnable sets of practices essential at all levels of healthcare. Individuals do not offer healthcare; instead, complex systems that function together, frequently involving vast numbers of people and organizations, do. Working in concert with large teams, including interprofessional collaboration, has become essential for 21st-century healthcare professionals, especially during this pandemic, to provide more effective and overall treatment for patients and the public.
- Building trust and cooperation with the public: medical professionals need to educate the masses to dispel speculation myths, curb vaccine hesitancy among youth, and counter the spread of false information across various social media platforms.
- Voting reforms and voter education: voting during elections during a pandemic like COVID-19 is a public health issue for India and the world. Yet voting is also a powerful tool to prevent future disease X. Educating voters about their choices and the need to vote for representatives supporting better pandemic preparedness can help elect pro-public health governments and policymakers.
- Protection to small business owners, self-employed groups, and single-parent households to maintain liquidity of such households: economic measures targeting households seek to reduce the impact of a disease X emergency like the COVID-19 pandemic on income and purchasing power. Self-employed workers have traditionally suffered during a pandemic due to ineligibility for unemployment insurance and not falling below the poverty level (the population is usually protected by the governments and given financial assistance). Quick income support for the self-employed, tax deferments as compensation for loss of revenue, or extending the social security benefits provided to other groups to this population are some measures that can be employed^49^. A crisis like a disease X pandemic affects all parts of the population. Still, its adverse impacts are felt more strongly by vulnerable social groups in any nation, for example, the poor, the elderly, the homeless, the unemployed, and children (especially those without adequate parental support). Poverty and inequality reduction systems and identifying gaps in the current social protection policies must be prioritized to protect this disadvantaged social fraction. A good example would be online classes for children during the COVID-19 pandemic to ensure continuous learning, financial support for the laid-off population, groceries, and other basic necessities for this vulnerable group^50^.
- The use of artificial intelligence (AI) tools like ChatGPT to create awareness about new Disease X outbreaks among travelers to endemic regions: ChatGPT, an innovation of OpenAI (San Francisco, CA, USA) can provide travelers with personalized information tailored to their area and time of travel and can be an essential tool in travel medicine as travelers are the most common carriers for converting an epidemic into a pandemic. AI tools like ChatGPT ensure that travelers are well-informed about potential health risks and the necessary precautions they must take while traveling, mainly in the face of a new Disease X outbreak. More recent developments in the field of AI can help in the future in case of a new novel outbreak like COVID-19.

## Conclusion

Disease X is a disease caused by a novel pathogen unknown to cause any disease in human beings. The world has seen many incidents of major disease outbreaks like the plague, the Spanish flu, and smallpox in the past, and swine flu, bird flu, dengue, and chikungunya virus epidemics during the present century, with COVID-19 being the most recent and severe disease. The lack of international cooperation, unequal distribution of healthcare knowledge and resources between high-income countries and middle-income countries, censorship of data regarding case numbers and deaths, leniency in social distancing measures, and the spread of false information among people about vaccination and drugs lead to the uncontrolled spread of the first disease X all over the world. India was relatively successful in controlling case numbers during the first wave; however, the second wave devastated the whole nation due to a more virulent strain, leniency of preventive measures, and mass gatherings for religious and political purposes. We recommend more research to study the spillover of viruses and other microbes from animals to humans, the development of mRNA vaccines, and a global iTPP to combat future disease X emergencies. Safety guidelines need to be set up and applicable beyond national borders, and intellectual property (like new sequences of disease X pathogen) between the developed and the developing or underdeveloped world needs to be shared. If we can carefully examine the mistakes made during the COVID-19 pandemic and use the lessons learned to implement a better prepandemic preparedness plan, we may be able to dodge another blow to the world’s human and economic resources caused by the ongoing coronavirus pandemic.

## Ethical approval

Ethical approval is not applicable for this narrative review article.

## Consent

Informed consent is not applicable for this narrative review article.

## Source of funding

Not applicable.

## Author contribution

M.B.: conceptualization, supervision, writing – original draft, and writing – review and editing; I.R., S.R., P.R., P.T., A.R.T., S.P., and M.B.: writing – original draft and writing – review and editing; A.A.: supervision and writing – review and editing. All authors are accountable for all the aspects of this work.

## Conflicts of interest disclosure

The authors declare no conflict of interest.

## Research registration unique identifying number (UIN)

1. Name of the registry: not applicable.
2. Unique identifying number or registration ID: not applicable.
3. Hyperlink to your specific registration (must be publicly accessible and will be checked): not applicable.

## Guarantor

Mainak Bardhan.

## Data availability statement

Not applicable.

## Provenance and peer review

Not commissioned, externally peer-reviewed.
